# A case of anastomotic stenosis of the small intestine caused by cholesterol crystal embolism

**DOI:** 10.1186/s40792-018-0442-3

**Published:** 2018-04-04

**Authors:** Koji Murono, Kazushige Kawai, Keisuke Hata, Shigenobu Emoto, Manabu Kaneko, Kazuhito Sasaki, Takeshi Nishikawa, Kensuke Otani, Toshiaki Tanaka, Masako Ikemura, Hiroaki Nozawa

**Affiliations:** 1 Department of Surgical Oncology, Faculty of Medicine, University of Tokyo, 7-3-1 Hongo, Bunkyo-ku, Tokyo, 113-8655 Japan; 2 Department of Pathology, Graduate School of Medicine, University of Tokyo, Tokyo, Japan

**Keywords:** Anastomotic stenosis, Cholesterol embolism, Intestinal stenosis

## Abstract

**Background:**

Cholesterol crystal embolism (CCE) is caused by small crystals of cholesterol dispersed from atherosclerotic plaques of the aorta. There is an increasing interest in CCE because of the increased use of endovascular treatments. Here, we report a rare case of intestinal stenosis caused by CCE after functional end-to-end anastomosis (FEEA). To our knowledge, this is the first report of CCE causing such an anastomotic stenosis.

**Case presentation:**

A 77-year-old male patient underwent laparoscopy-assisted low anterior resection and protective ileostomy for rectal carcinoid tumor. He was admitted to our hospital with ileus 1 year after stoma closure. Eosinophils and creatine kinase level were slightly elevated. Computed tomography revealed a stricture with thickened intestinal wall just distal to the anastomosis site of the ileostomy. The wall of the descending aorta appeared shaggy due to thrombosis. The patient underwent laparoscopic small-bowel resection because ileus reoccurred after any oral intake.

Histopathological findings of the resected specimen showed fibrotic changes distal to the anastomosis site, and needle-shaped cholesterol embolus was observed in the submucosal layer. Thus, the stenosis was considered to be caused by CCE.

**Conclusion:**

This appears to be the first published report of stenosis due to CCE at such an anastomotic site. Intestinal CCE is difficult to diagnose preoperatively and is associated with poor prognosis. If eosinophilia is present or shaggy aorta is observed, CCE should be suspected to make correct diagnosis and prevent recurrence of CCE.

## Background

Cholesterol crystal embolism (CCE) was first reported by Mulliken and Bartlett [1]. Small crystals of cholesterol disseminated from atherosclerosis of the aorta induce inflammation, intimal thickening, and occlusion of small arteries [2]. CCE may cause exanthema (also known as “blue toe”), chronic renal failure, cerebral infarction, and other conditions [3].

Endovascular treatment such as cardiac catheterization, antithrombotic treatment, and anticoagulation therapies increase the risk for CCE. There is increasing interest in CCE because of the increased use of endovascular treatments following introduction of low-stress techniques such as the endovascular aneurysm repair (EVAR). In one study, 2% of the patients who underwent EVAR developed embolic complications such as renal dysfunction, blue toe syndrome, and ischemic colitis [4]. Notably, the complication rate was 25% in cases presenting with a shaggy aorta [5].

CCE sometimes causes intestinal ischemia, which may result in perforation or stenosis [1]. Here, we report a rare case of intestinal stenosis caused by CCE after functional end-to-end anastomosis (FEEA). To our knowledge, this is the first report of CCE causing such an anastomotic stenosis.

## Case presentation

**Fig. 1:**
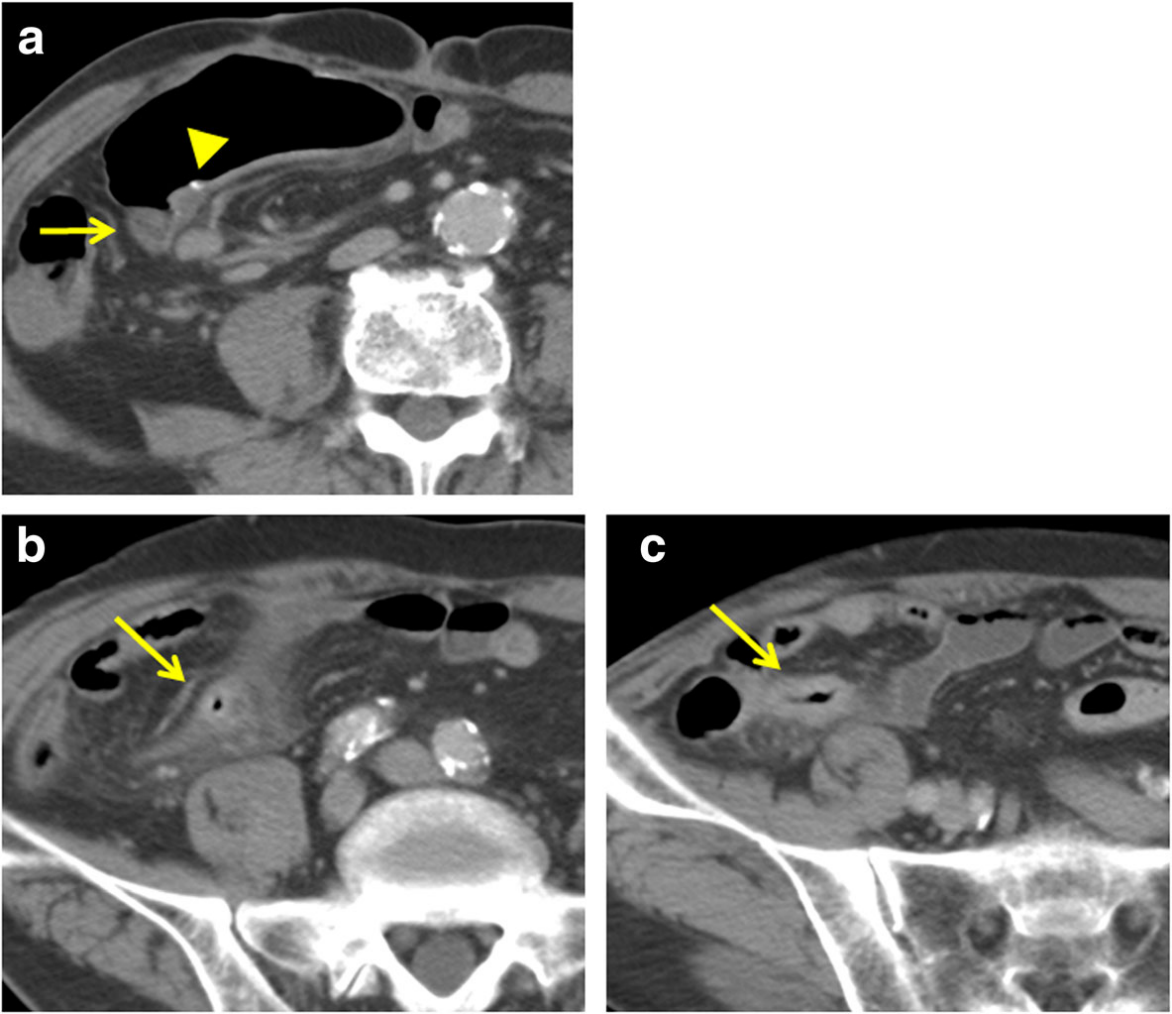
**a** Computed tomography (CT) scan of the small intestine, revealing diameter change on the distal side of the anastomotic site (arrow). The triangle indicates a staple of the functional end-to-end anastomosis. **b**, **c** Wall thickening and inflammation of the mesentery were observed at the stenotic site (arrow)

**Fig. 2:**
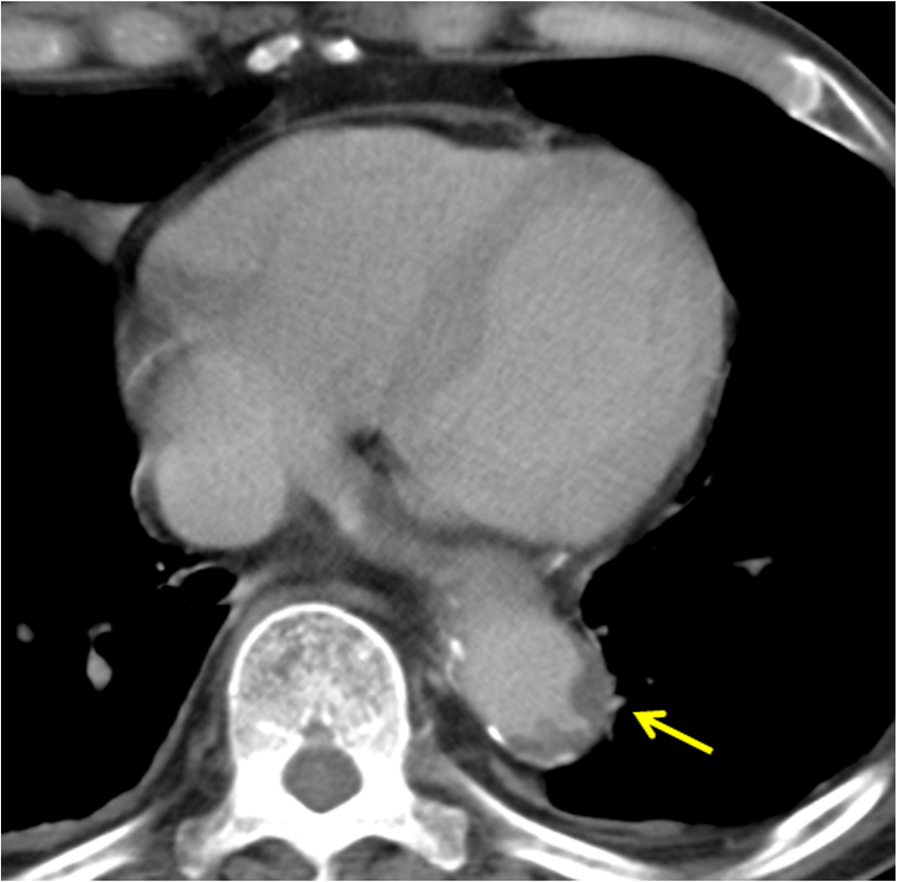
Computed tomography (CT) scan revealing shaggy aortic wall due to atherosclerosis (arrow)

**Fig. 3:**
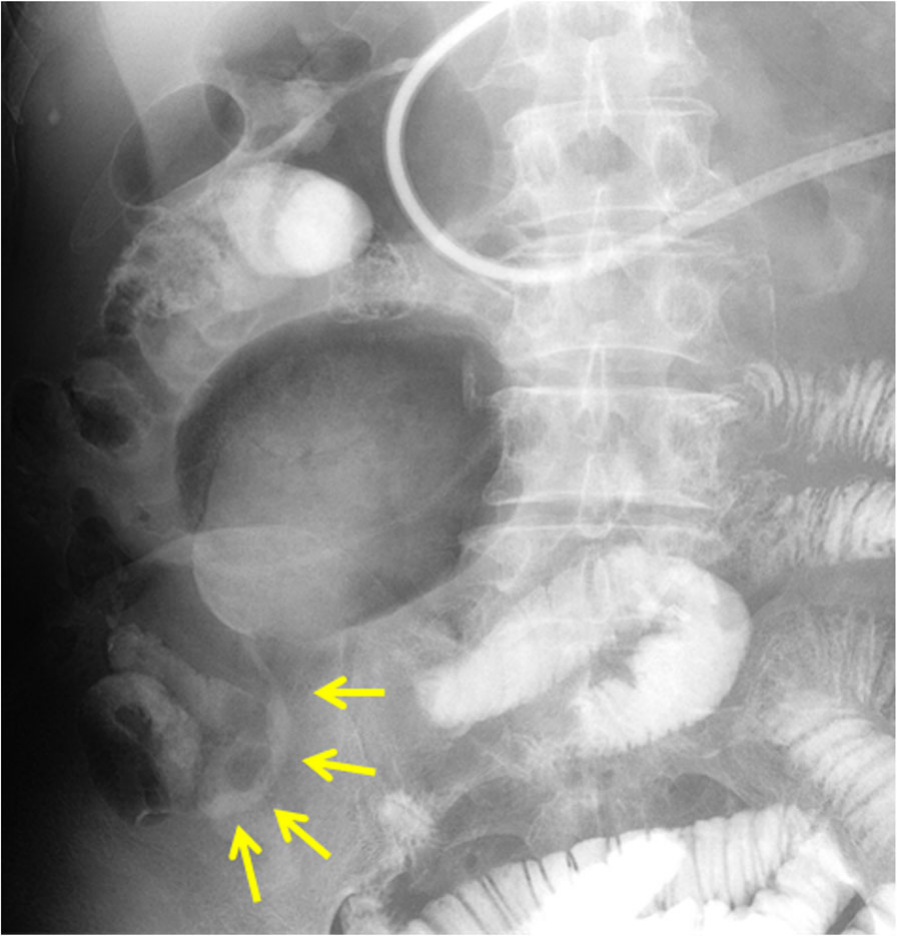
Small-bowel series revealing stenosis of the small intestine (arrow) and dilation of the proximal side. The length of the stenosis was about 5 cm

A 77-year-old male patient underwent laparoscopy-assisted low anterior resection and protective ileostomy for rectal carcinoid tumor. The ileostomy closure was performed after 6 months; FEEA was performed for intestinal reconstruction. One year after stoma closure, the patient was admitted to our hospital complaining of abdominal pain. He was a smoker and on medication for hypertension. He had not become hyperlipidemic. There was a slight elevation in the number of eosinophils (8.0%, normal range 1.0–5.0%) and creatine kinase level (1.35 mg/dl, normal range 0.6–1.2). Computed tomography (CT) scan revealed a region of dilated small intestine around the FEEA site and a change in the diameter on the distal side, adjacent to the anastomosis. The intestinal wall of the stenotic site was thickened and there was an increase in the CT signal from the local mesentery, indicating inflammation (Fig. 1). There was no stenosis or occlusion of the mesenteric artery or vein. The wall of the descending aorta was shaggy due to thrombosis (Fig. 2). The length of the stenosis as seen on the small bowl series was about 5 cm (Fig. 3). Although a long tube insertion improved the symptoms immediately, abdominal pain and vomiting recurred after oral intake. Therefore, we performed a laparoscopic small-bowel resection with reconstruction of the small bowel via FEEA.

**Fig. 4:**
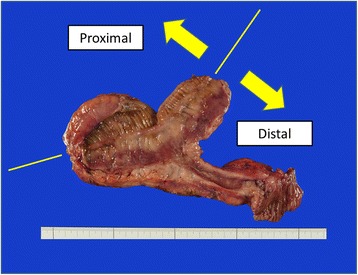
Macroscopic findings of the resected specimen. The line indicates the original anastomotic site of the closed ileostomy. The intestinal wall was thickened and the Kerckring folds were absent distal to the anastomosis site. Ischemic change was not observed at the distal margin of the resected specimen

**Fig. 5:**
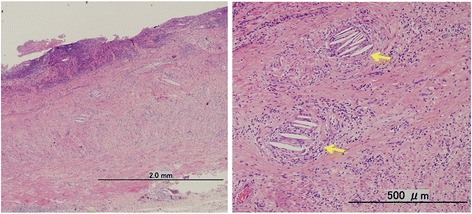
Histopathological findings revealed fibrotic changes to the whole bowel wall, and needle-shaped cholesterol crystals were observed in the submucosal layer of the stenotic site (arrow)

Macroscopic findings of the resected specimen revealed that the intestinal wall was thickened and the Kerckring folds were absent for a length of 7 cm, beginning on the distal side, adjacent to the anastomosis site (Fig. 4). Histopathological findings showed ulcerative scars and fibrotic changes at the distal side of the anastomosis, and needle-shaped cholesterol crystals were observed in the submucosal layer (Fig. 5). Although the hospital stay was prolonged because of the course of antibiotics required to treat a postoperative abscess, he was discharged on postoperative day 33.

### Discussion

CCE, induced by cholesterol crystals dispersed from atherosclerotic plaques of the aorta [2], may cause chronic renal failure, blue toe, livedo reticularis, and intestinal ischemia with perforation, stenosis, or inflammatory mass formation [1, 6, 7]. The most frequent site of intestinal CCE is the large intestine, followed by the small intestine, and the stomach [8]. In the present case, anastomotic stenosis occurred because of the ischemic change at the distal side of the anastomosis. As there are no prior reports of stenosis at such an anastomotic site, it is unknown whether bowel resection is a risk factor for CCE. The ischemic change in our case was limited to the distal side of the anastomosis. The connection between the proximal and distal side of the anastomosis has been eliminated by dissecting the mesenteric marginal artery at the time of FEEA. We speculate, therefore, that cholesterol emboli were trapped regionally on the distal side of the anastomosis without spreading, which induced anastomotic stenosis.

CCE is mainly diagnosed using histopathological examination. The diagnosis is confirmed by the presence of cholesterol crystals in tissue biopsy samples. Tissue biopsy sampling is not necessary in the case of the presence of the classic triad, that is, atherosclerotic disease, acute or subacute renal failure, and typical skin findings such as blue toe [9]. In the present case, CCE was diagnosed based on the resected specimen. The anastomotic site became fibrotic with inflammation and cholesterol crystals were observed. Therefore, the stenosis was considered to be caused by an ischemic change due to CCE. It is difficult to diagnose intestinal CCE preoperatively because the symptoms are nonspecific. Moreover, the intestinal clinical condition is similar to that of nonocclusive mesenteric ischemia because the cholesterol crystals are very small and mesenteric arteries are not usually occluded [10, 11]. Elevated eosinophil levels and renal failure raise the suspicion of CCE [9]. In the case of intestinal stenosis with inflammation, eosinophil count and urine protein should be examined as a less invasive method [12]. Although “blue toe” was not observed in the present case, examination of the feet may contribute to the diagnosis of CCE.

Treatment of CCE mainly comprises of supportive care, including pain control, blood pressure management, and dialysis [7]. Although corticosteroids and low-density lipoprotein apheresis may reduce the recurrence of CCE [13, 14], the benefit of this therapy has not been totally established [7]. Antiplatelet therapy or anticoagulation therapy does not prevent CCE; these therapies rather increase the risk [7]. Recently, statins have been suggested to be effective in preventing CCE because of regression and stabilization of plaques in the aorta [15, 16]. CCE is likely to recur and the recurrence is associated with poor prognosis [17]. Therefore, once the diagnosis of CCE is confirmed, even in patients without hyperlipidemia, statins should be started in addition to cessation of antiplatelet and anticoagulant therapy.

## Conclusions

We report a rare case of intestinal anastomotic stenosis due to CCE. Intestinal CCE is difficult to diagnose preoperatively and is associated with poor prognosis. When eosinophilia or shaggy aorta is observed, CCE should be suspected to reach an accurate diagnosis and reduce the risk of recurrence.

## Abbreviations

CCE: Cholesterol crystal embolism
CT: Computed tomography
EVAR: Endovascular aneurysm repair
FEEA: Functional end-to-end anastomosis
